# Serial Section Array Scanning Electron Microscopy Analysis of Cells from Lung Autopsy Specimens following Fatal A/H1N1 2009 Pandemic Influenza Virus Infection

**DOI:** 10.1128/JVI.00644-19

**Published:** 2019-09-12

**Authors:** Michiyo Kataoka, Kinji Ishida, Katsutoshi Ogasawara, Takayuki Nozaki, Yoh-Ichi Satoh, Tetsutaro Sata, Yuko Sato, Hideki Hasegawa, Noriko Nakajima

**Affiliations:** a Department of Pathology, National Institute of Infectious Diseases, Tokyo, Japan; b Technical Support Center for Life Science Research, Iwate Medical University, Iwate, Japan; c Department of Medical Education, Iwate Medical University, Iwate, Japan; d Global Virus Network, Baltimore, Maryland, USA; Icahn School of Medicine at Mount Sinai

**Keywords:** autopsy, electron microscopy, influenza virus

## Abstract

A/H1N1 2009 pandemic influenza virus (A/H1N1/pdm09) was first identified as a novel pandemic influenza A virus (IAV) in 2009. Previously, we reported that many viral antigens were detected in type II alveolar epithelial cells (AEC-IIs) within autopsied lung tissue from a patient with A/H1N1/pdm09 pneumonia. It is important to identify the association between the virus and host cells to elucidate the pathogenesis of IAV pneumonia. To investigate the distribution of virus particles and morphological changes in host cells, the autopsied lung specimens from this patient were examined using transmission electron microscopy (TEM) and a novel scanning electron microscopy (SEM) method. We focused on AEC-IIs as viral antigen-positive cells and on monocytes/macrophages (Ms/Mϕs) and neutrophils (Neus) as innate immune cells. We identified virus particles and intranuclear dense tubules, which are associated with matrix 1 (M1) proteins from IAV. Large-scale two-dimensional observation was enabled by digitally “stitching” together contiguous SEM images. A single whole-cell analysis using a serial section array (SSA)-SEM identified virus particles in vesicles within the cytoplasm and/or around the surfaces of AEC-IIs, Ms/Mϕs, and Neus; however, intranuclear dense tubules were found only in AEC-IIs. Computer-assisted processing of SSA-SEM images from each cell type enabled three-dimensional (3D) modeling of the distribution of virus particles within an ACE-II, a M/Mϕ, and a Neu.

**IMPORTANCE** Generally, it is difficult to observe IAV particles in postmortem samples from patients with seasonal influenza. In fact, only a few viral antigens are detected in bronchial epithelial cells from autopsied lung sections. Previously, we detected many viral antigens in AEC-IIs from the lung. This was because the majority of A/H1N1/pdm09 in the lung tissue harbored an aspartic acid-to-glycine substitution at position 222 (D222G) of the hemagglutinin protein. A/H1N1/pdm09 harboring the D222G substitution has a receptor-binding preference for α-2,3-linked sialic acids expressed on human AECs and infects them in the same way as H5N1 and H7N9 avian IAVs. Here, we report the first successful observation of virus particles, not only in AEC-IIs, but also in Ms/Mϕs and Neus, using electron microscopy. The finding of a M/Mϕ harboring numerous virus particles within vesicles and at the cell surface suggests that Ms/Mϕs are involved in the pathogenesis of IAV primary pneumonia.

## Abstract (precis)

Generally, it is difficult to observe IAV particles in postmortem samples from patients with seasonal influenza. In fact, only a few viral antigens are detected in bronchial epithelial cells from autopsied lung sections. Previously, we detected many viral antigens in AEC-IIs from the lung. This was because the majority of A/H1N1/pdm09 in the lung tissue harbored an aspartic acid-to-glycine substitution at position 222 (D222G) of the hemagglutinin protein. A/H1N1/pdm09 harboring the D222G substitution has a receptor-binding preference for α-2,3-linked sialic acids expressed on human AECs and infects them in the same way as H5N1 and H7N9 avian IAVs. Here, we report the first successful observation of virus particles, not only in AEC-IIs, but also in Ms/Mϕs and Neus, using electron microscopy. The finding of a M/Mϕ harboring numerous virus particles within vesicles and at the cell surface suggests that Ms/Mϕs are involved in the pathogenesis of IAV primary pneumonia.

## INTRODUCTION

Influenza A viruses (IAVs) are enveloped viruses that contain a segmented negative-strand RNA genome. IAVs express the spike glycoproteins hemagglutinin (HA) and neuraminidase (NA) on the viral surface (1, 2). HA proteins of human seasonal IAVs bind to α-2,6-linked sialic acid, which is expressed abundantly on epithelial cells lining the human respiratory tract (except the alveoli) (3). Therefore, seasonal IAVs rarely infect alveolar epithelial cells (AECs). A/H1N1 2009 pandemic influenza virus (A/H1N1/pdm09) was the causative IAV of a novel influenza pandemic in 2009 (4). Since then, A/H1N1/pdm09 has continued to circulate as a seasonal virus, along with A/H3N2 influenza virus. IAVs accumulate point mutations during replication, thereby giving rise to quasispecies. One of these mutations results in changes to the HA amino acid sequence. Since the first appearance of A/H1N1/pdm09, an amino acid substitution from aspartic acid to glycine at position 222 (D222G) in the HA protein has been identified sporadically (5–7). The D222G substitution alters the receptor-binding preference of HA so that it binds to α-2,3-linked sialic acids, which are expressed abundantly on human AECs; therefore, this virus has the potential to cause viral pneumonia (5–7).

Previously, we reported a fatal case of A/H1N1/pdm09 infection (8). The results of next-generation sequencing revealed that a majority of the viruses detected in the lung carried the D222G substitution in HA (9). The patient presented with primary viral pneumonia, which developed into severe acute respiratory distress syndrome (ARDS), resulting in death from respiratory failure within 7 days of disease onset (8). Histopathological examination of the lung revealed diffuse alveolar damage, along with the presence of many viral antigens, genomic RNA, and mRNA in AECs (8, 10, 11). In this case, a large number of virus particles remained in the tissue at the time of death; thus, virus particles were observed in lung specimens by transmission electron microscopy (TEM) (8).

Here, we analyzed the same lung specimens using a novel scanning electron microscopy (SEM) method in addition to TEM (12). Whereas conventional SEM does not require embedding and sectioning steps before imaging and observes only the surfaces of specimens, the novel SEM technique used in this study involves mounting single or serial ultrathin sections on a glass slide and staining them with heavy metals, thereby enabling imaging at a resolution comparable with that of TEM by recording backscattered electrons. By digitally “stitching” together contiguous SEM images, a large-scale two-dimensional (2D) image can be obtained at high resolution (13). Serial section array (SSA)-SEM analyzes each cross-sectional image throughout a single whole cell to reveal the distribution of specific structures (12, 14, 15). After manual tracing of specific structures, such as virus particles, cytoplasm, and nucleus, in each serial section, the structures are visualized in a three-dimensional (3D) model (12, 14, 15). We focused on type II alveolar epithelial cells (AEC-IIs) as virus antigen-positive cells and on monocytes/macrophages (Ms/Mϕs) and neutrophils (Neus) as immune cells that respond to influenza virus infection. We used TEM and SEM to examine the distribution of influenza virus particles and matrix 1 (M1) protein-associated intranuclear dense tubules (16–20) within these cell types in specimens of autopsied lung in which A/H1N1/pdm09 harboring the D222G substitution (A/H1N1/pdm09-D222G) had proliferated. Here, even though we were able to examine only a single human case, we present the first successful observation of influenza virus particles and M1-associated intranuclear dense tubules in large-scale 2D images and 3D images.

## RESULTS

### TEM imaging of type II alveolar epithelial cells.

**FIG 1:**
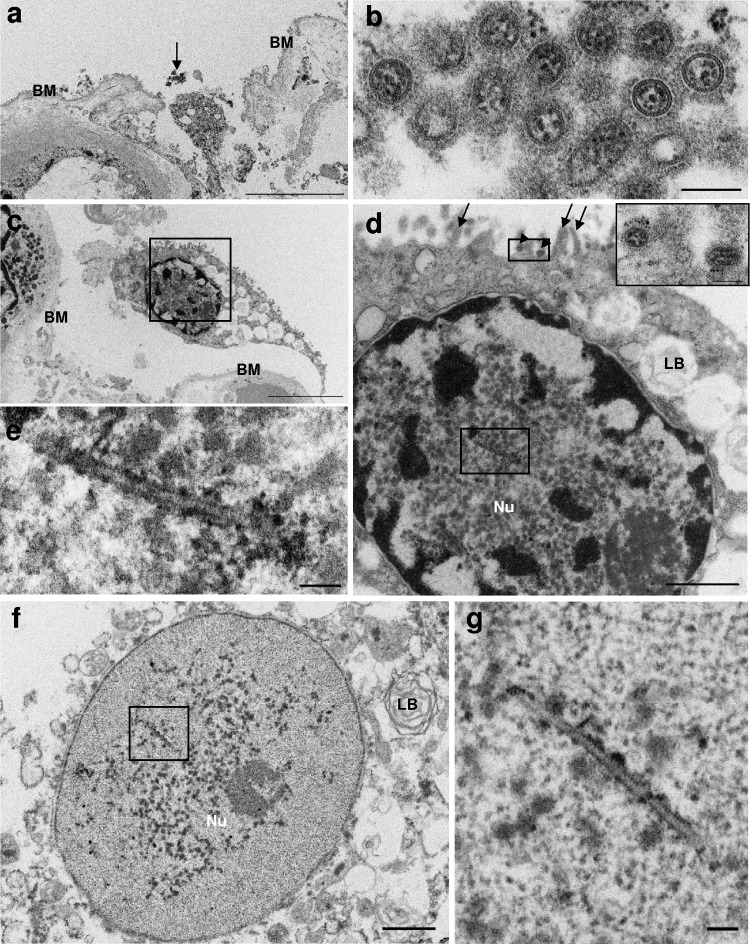
(a) TEM images revealing virus particles in the alveolar space. Scale bar = 5 μm. (b) Higher-magnification images of the area indicated by the arrow in panel a. The virus particles show internal nucleocapsids surrounded by an envelope with prominent spikes. Scale bar = 100 nm. (c) AEC-II desquamated from the basement membrane. Scale bar = 5 μm. (d) Higher-magnification images of the boxed area in panel c. The arrowheads indicate virus particles, and the arrows indicate microvilli. Dense tubules are observed in the nuclei of AEC-II (boxed). Scale bar = 1 μm. (Inset) Virus particles with high-electron-density nucleocapsids and spikes. Scale bars = 100 nm. (f) Dense tubules are also observed in the denatured nucleus of AEC-II. Scale bar = 1 μm. (e and g) Dense tubular structures in the nucleus (boxed areas from panels d and f, respectively). Scale bars = 100 nm. BM, basement membrane; Nu, nucleus; LB, lamellar body.

Most of the AEC-IIs observed in the lung tissue specimen prepared for electron microscopy (EM) observation were desquamated from the basement membrane; 50 TEM images of AEC-IIs were analyzed. Virus particles were found in the alveolar space and around AEC-IIs (Fig. 1a and c). The virus particles were about 95 nm in diameter and surrounded by glycoprotein spikes and envelopes; in addition, they contained virus ribonucleoprotein (RNP) complexes (Fig. 1b). Although no budding virus particles were identified, 6/50 TEM images of AEC-IIs revealed virus particles around the cell surface (Table 1). In these images, virus particles could be differentiated from microvilli (Fig. 1d). The virus particles were characterized by high-electron-density RNPs surrounded by glycoprotein spikes (Fig. 1d, inset). Notably, dense helical tubules were observed in the nucleus (Fig. 1d and e); these intranuclear tubules have been reported in IAV-infected cells and identified as an M1 protein-associated structure (16–20). We also observed these structures in the degenerated nuclei of AEC-II cells (Fig. 1f and g). Intranuclear dense tubules were identified in 23/50 TEM images (Table 1). The tubules were 108 to 698 nm in length, with a diameter of 22 to 39 nm.

**TABLE 1:** Numbers of TEM images containing specific features

Cell type	Total no. of images	No. of images with:			
		Virus around cell surface^*a*^	Intravesicular virus	Intranuclear dense tubules	Budding virus
AEC-II	50	6	1	23	0
M/Mϕ	30	3	4	0	0
Neu	30	6	0	0	0

a Within 2 μm from the cell surface.

### TEM imaging of monocytes/macrophages.

**FIG 2:**
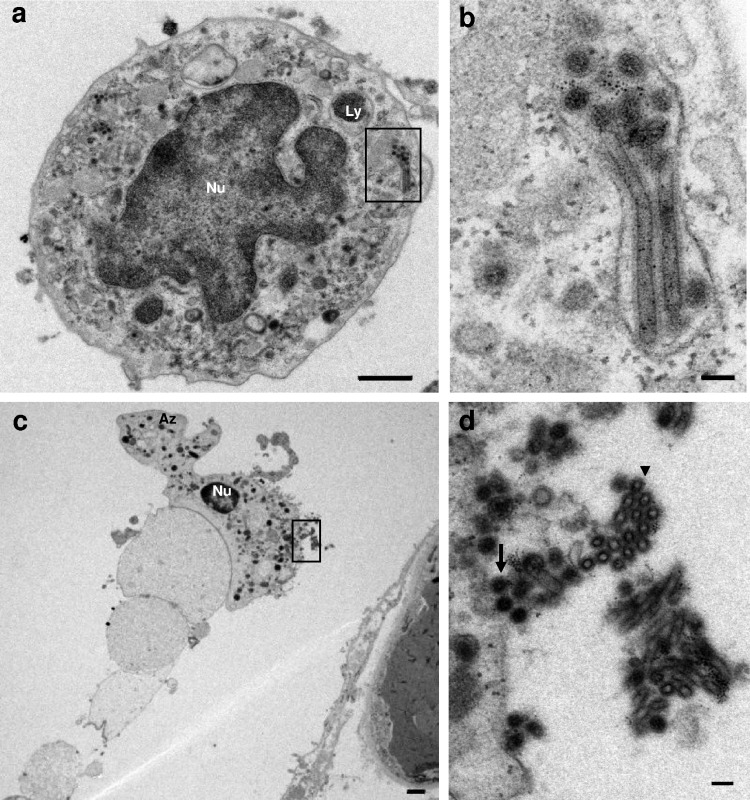
M/Mϕ and Neu in the alveolar lumen. (a) Virus particles in a vesicle within the cytoplasm of a M/Mϕ. Scale bar = 1 μm. (b and d) Boxed areas from panels a and c, respectively, at higher magnification. (b) Filamentous and spherical virus particles within a vesicle. Scale bar = 100 nm. (c) A Neu associated closely with a denatured type II alveolar epithelial cell. Scale bar = 1 μm. (d) Virus particles around the cell surface. The arrow indicates nucleocapsids (mean diameter, 94 nm), and the arrowhead indicates hollow particles (mean diameter, 68 nm). Scale bar = 100 nm. Nu, nucleus; Ly, lysosome; Az, azurophilic granule.

Ms/Mϕs were less common than AEC-IIs; therefore, 30 TEM images of Ms/Mϕs were analyzed. In four of them, several filamentous and spherical virus particles were observed within cytoplasmic vesicles (Fig. 2a and b and Table 1). The mean diameters of the filamentous and spherical virus particles were 75 nm and 95 nm, respectively. Also, the longest filamentous virus was 676 nm. The observation that the diameter of a filamentous virus was less than that of a spherical virus is consistent with the results of a previous report (21). No budding virus particles or intranuclear dense tubules were observed in any of the 30 images.

### TEM imaging of neutrophils.

Neus were present in numbers similar to those of Ms/Mϕs; therefore, 30 TEM images of Neus were analyzed. In three of them, Neus were closely associated with desquamated and degenerated AEC-IIs, suggesting the occurrence of phagocytosis (Fig. 2c). Six images showed virus particles around the cell surface (Table 1). Some of these virus particles contained nucleocapsids (mean diameter, 94 nm), whereas others were hollow (mean diameter, 68 nm) (Fig. 2d). No budding virus particles, virus particles in vesicles, or intranuclear dense tubules were observed in any of the 30 TEM images.

### Stitching together SEM images of an alveolus.

**FIG 3:**
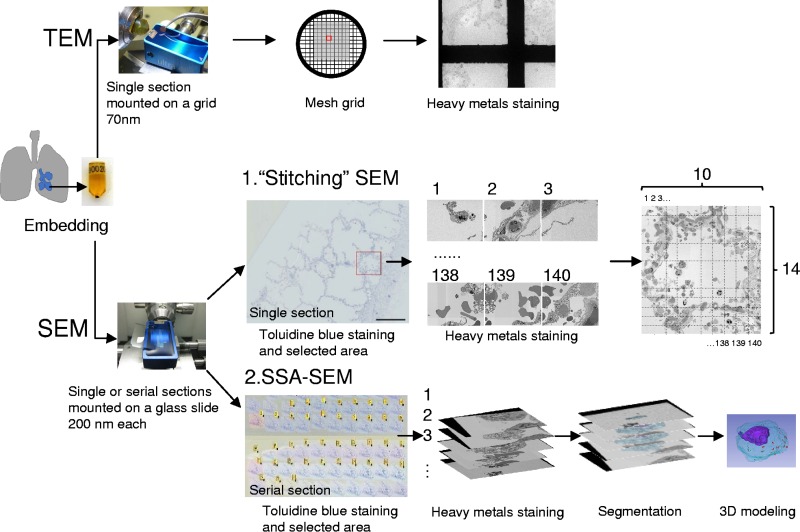
Comparative illustration of TEM and SEM workflows. Small pieces of fixed lung tissue were embedded in epoxy resin. Ultrathin sections 70 nm thick were mounted on a grid and stained with heavy metals for TEM. A novel SEM method, stitching together of contiguous SEM and SSA-SEM images, is shown. Ultrathin single or serial sections (each 200 nm thick) were mounted on a conductive glass slide and stained with toluidine blue for light microscopy. After staining with heavy metal, a 10-by-14 array of contiguous SEM images was digitally stitched together to generate an entire alveolus at high resolution, sufficient to confirm the presence of virus particles. For SSA-SEM images, sections that included the objective cells were colored yellow and stained with heavy metal prior to SEM. The SSA-SEM images had to be aligned for 3D reconstruction. Subsequently, the cytoplasm, nuclei, and viruses were segmented by tracing their boundaries. The images were then imported into the 3D software and visualized in a 3D model.

**FIG 4:**
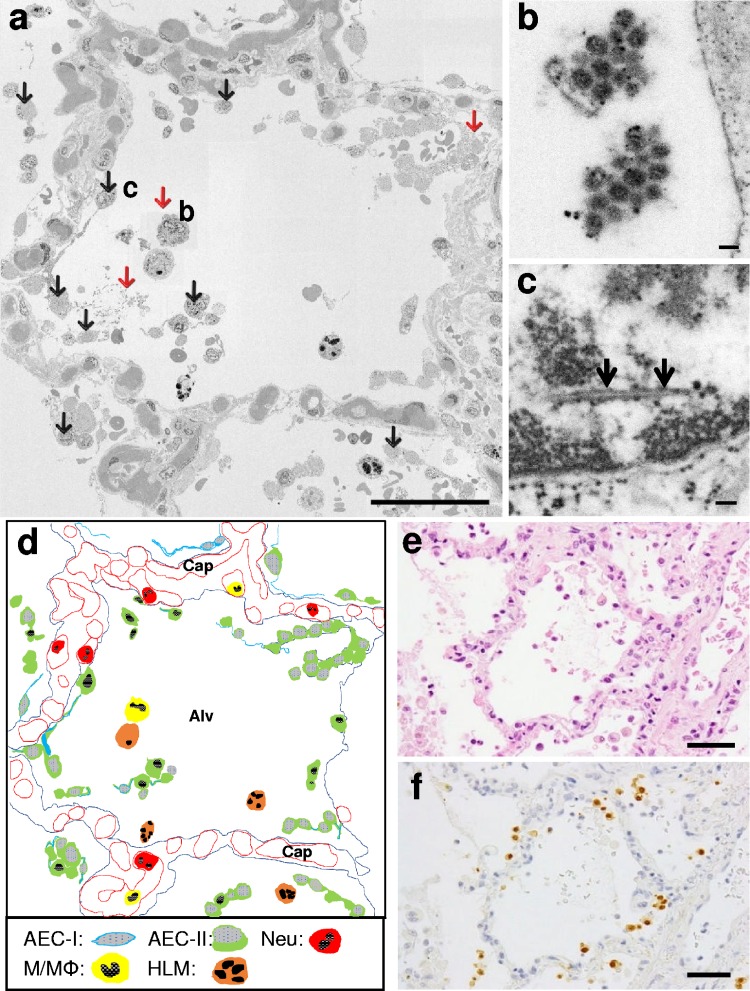
Stitching together contiguous SEM images of an entire alveolus. (a) Virus particles (red arrows) and dense tubules (black arrows) observed in the composite image after stitching. Scale bar = 50 μm. (b and c) Virus particles and dense tubules (arrows) from areas b and c in panel a, respectively, at higher magnification. Scale bars = 100 nm. (d) Schema of panel a. AEC-Is are thin and contain less cytoplasm, whereas AEC-IIs are characterized by lamellar bodies. The nucleus containing chromatin is shown as black, and the denatured nucleus is gray. Most of the AEC-Is and AEC-IIs are detached from the basement membrane. Neus and Ms/Mϕs are also found in capillaries (Cap). Hemosiderin-laden macrophages (HLMs) are observed in the alveolar lumen (Alv). (e and f) Serial sections of a different alveolus with a level of inflammation similar to that shown in panel a. (e) Hematoxylin-eosin staining. Scale bar = 50 μm. (f) Immunohistochemistry showing AEC-IIs stained positive (brown) for influenza virus nucleoprotein. Scale bar = 50 μm.

An entire alveolus is too large (in this example, 209 by 206 μm) to be presented in cross-section in a single TEM or SEM image. Therefore, we built a set of contiguous SEM images and digitally stitched them together to generate a cross-section of an entire alveolus at high resolution. Briefly, a single 200-nm-thick section was mounted on a glass slide and stained with toluidine blue to facilitate selection of an appropriate region by light microscopy. The section was then stained with heavy metals, thereby enabling a 10-by-14 array of contiguous SEM images to be taken and digitally stitched together (Fig. 3). In the alveolus shown in Fig. 4, type I and type II AECs and Ms/Mϕs are observed in the alveolar lumen. Neus and Ms/Mϕs are also present in a capillary blood vessel. The patient suffered chronic heart failure, and hemosiderin-laden macrophages were present in the alveolar lumen. Desquamated AEC-IIs had little visible chromatin in the expanded nucleus. At low magnification, the stitched SEM image was similar to the optical microscopic image stained with hematoxylin-eosin (Fig. 4a and e); however, the stitched SEM image could be highly magnified using virtual-slide software (NDP.view2) so that virus particles (Fig. 4b) and intranuclear dense tubules (Fig. 4c) could be visualized. Virus particles were present at three extracellular sites, and intranuclear tubular structures were found in eight AEC-IIs in the alveolus (Fig. 4a). In another alveolus with a similar level of inflammation, immunohistochemistry revealed that the distribution of AEC-IIs positive for the nucleoprotein of A/H1N1/pdm09 (Fig. 4f) was similar to that of AEC-IIs harboring intranuclear dense tubules (Fig. 4a).

### SSA-SEM imaging of type II alveolar epithelial cells.

SSA-SEM analysis revealed the distribution of specific structure throughout a single cell. Briefly, 100 serial sections (each 200 nm thick) were mounted on a platinum-palladium-coated glass slide and stained with toluidine blue. Then, sections containing the object cells were marked in yellow. Next, each marked serial section was captured by SEM after heavy metal staining (Fig. 3). Six AEC-IIs, six Ms/Mϕs, and three Neus were analyzed. The numbers of SSA-SEM images containing specific features are shown in Table 2, and the numbers of virus particles and intranuclear dense tubules in each SSA-SEM image are shown in Table 3.

**TABLE 2:** Numbers of SSA-SEM images containing specific features in each cell

Cell type	Total no. of images	No. of images with:		
		Virus around cell surface^*d*^	Intravesicular virus	Intranuclear dense tubules/nuclei
AEC-IIs				
AEC-II-1	42	36	0	16/18
AEC-II-2	50	27	0	15/30
AEC-II-3	50	0	0	0/37
AEC-II-4	34	0	0	0/22
AEC-II-5	61	0	0	13/27
AEC-II-6^*a*^	41	14	15	16/19
Total	278	77	15	60/153
Ms/Mϕs				
M/Mϕ-1	38	1	14	0/34
M/Mϕ-2^*b*^	37	31	25	0/22
M/Mϕ-3	45	23	22	0/29
M/Mϕ-4	35	2	14	0/24
M/Mϕ-5	35	0	0	0/29
M/Mϕ-6	36	0	0	0/20
Total	226	57	75	0/158
Neus				
Neu-1^*c*^	38	13	16	0/30
Neu-2	31	1	0	0/22
Neu-3	31	0	0	0/26
Total	100	14	16	0/78

a See Fig. 5.

b See Fig. 6.

c See Fig. 7.

d Within 2 μm from the cell surface.

**TABLE 3:** Total numbers of virus particles and intranuclear dense tubules in SSA-SEM images from each cell

Cell type^*e*^	No. around cell surface^*d*^		No. intravesicular			No. with intranuclear dense tubules
	Spherical/oval virus	Filamentous virus	Spherical/oval virus	Filamentous virus	Maximum in one vesicle	
AEC-II-1	365	0	0	0	0	161
AEC-II-2	146	0	0	0	0	93
AEC-II-3	0	0	0	0	0	0
AEC-II-4	0	0	0	0	0	0
AEC-II-5	0	0	0	0	0	76
AEC-II-6^*a*^	54	0	58	0	7	160
M/Mϕ-1	5	0	225	0	39	0
M/Mϕ-2^*b*^	1,904	180	585	53	22	0
M/Mϕ-3	105	2	587	2	36	0
M/Mϕ-4	23	0	294	6	16	0
M/Mϕ-5	0	0	0	0	0	0
M/Mϕ-6	0	0	0	0	0	0
Neu-1^*c*^	97	0	108	0	13	0
Neu-2	3	0	0	0	0	0
Neu-3	0	0	0	0	0	0

a See Fig. 5.

b See Fig. 6.

c See Fig. 7.

d Within 2 μm from the cell surface.

e No statistically significant differences in the numbers of virus particles in the vesicles and around the cell surfaces between AEC-IIs and Ms/Mϕs by Mann-Whitney U test.

**FIG 5:**
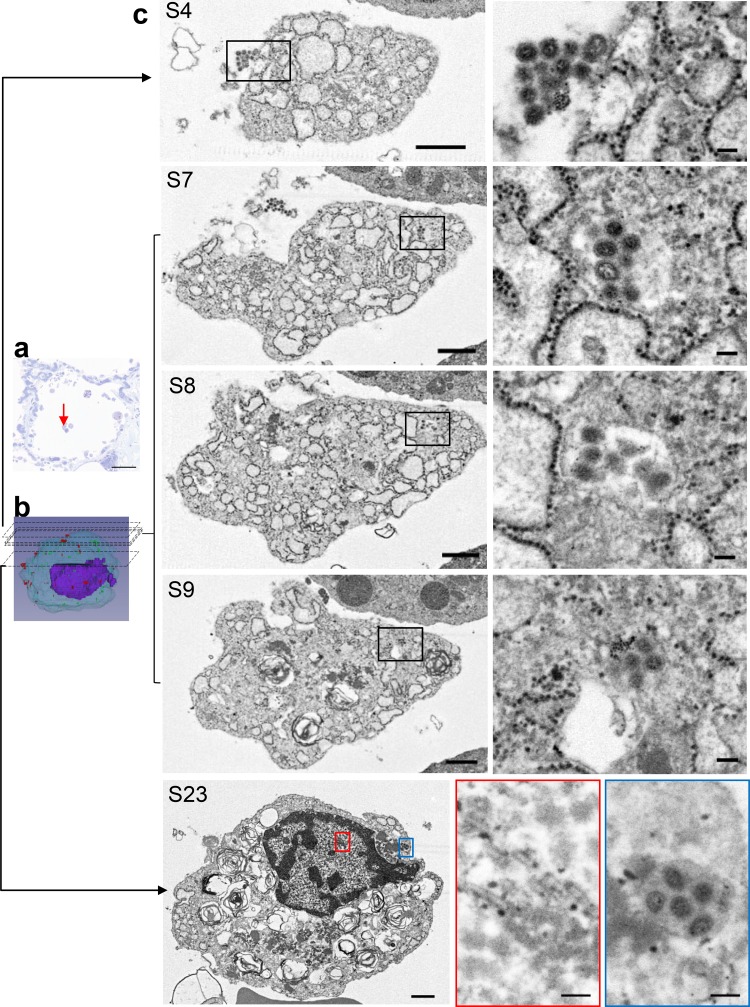
A single desquamated AEC-II in the alveolar space was analyzed by SSA-SEM. (a) Light microscopy of an alveolar section stained with toluidine blue showing the location of an AEC-II (red arrow). Scale bar = 50 μm. (b) Static 3D image of AEC-II-6. (c) Representative sections (S4, S7 to S9, and S23) from 41 consecutive sections (each 200 nm thick) of AEC-II-6. The boxed areas (left) are shown at higher magnification on the right. S4 shows virus particles around the cell, and S7 to S9 show vesicles containing several virus particles. S23 shows both intranuclear dense tubules (red box) and intravesicular virus particles (blue box). (Left) Scale bars = 1 μm. (Right) Scale bars = 100 nm.

Six randomly selected AEC-IIs were analyzed by SSA-SEM. All were desquamated from the basement membrane. Virus particles were observed around the surfaces of three of six AEC-IIs (Table 2). Intravesicular virus particles were observed only in AEC-II-6, and all were spherical/oval (Tables 2 and 3 and Fig. 5). Intranuclear dense tubules were observed in four of six AEC-IIs. Although it is possible that virus particles around 100 nm in diameter might be missed by analyzing serial sections that were 200 nm thick, neither virus particles nor dense tubules were observed in AEC-II-3 and AEC-II-4 (Table 2).

### SSA-SEM imaging of monocytes/macrophages.

**FIG 6:**
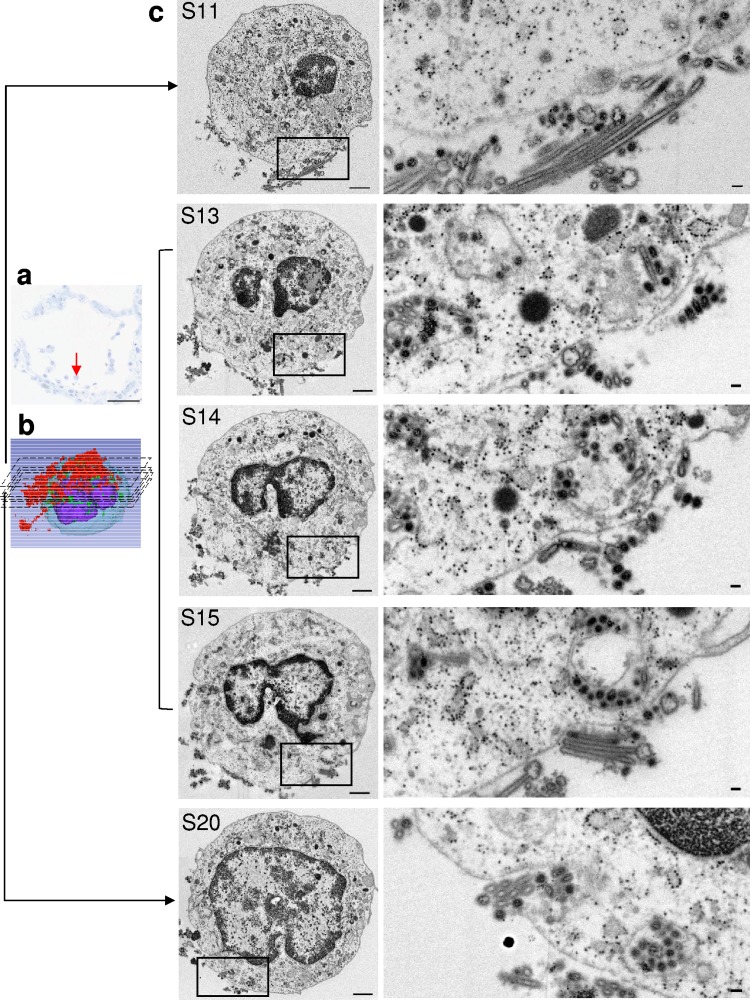
A single whole M/Mϕ in the alveolar space was analyzed by SSA-SEM. (a) Light microscopy of an alveolar section stained with toluidine blue showing the location of a M/Mϕ (red arrow). Scale bar = 50 μm. (b) Static 3D image of M/Mϕ-2. (c) Representative sections (S11, S13 to S15, and S20) from 37 consecutive sections (each 200 nm thick) of M/Mϕ-2. The boxed areas (left) are shown at higher magnification on the right. S13 to S15 show a vesicle that contains several virus particles. Spherical and filamentous virus particles are seen around the cell surface and in the vesicle (S11, S13 to S15, and S20). Multiple virus particles are observed in the extracellular region in which the cell membrane enters the cytoplasm (S20). (Left) Scale bars = 1 μm. (Right) Scale bars = 100 nm.

Six randomly selected Ms/Mϕs were analyzed by SSA-SEM (Tables 2 and 3). M/Mϕ-2 harbored numerous virus particles in spherical, oval, and filamentous forms; they were observed around the cell surface and in vesicles (Table 3 and Fig. 6b). Intravesicular virus particles were observed in about half of the SSA-SEM images of four Ms/Mϕs (M/Mϕ-1, -2, -3, and -4). Intranuclear dense tubules were not found in any of the 226 SSA-SEM images from six whole cells (Table 2). Neither virus particles nor dense tubules were observed in M/Mϕ-5 and M/Mϕ-6 (Table 2).

### SSA-SEM imaging of neutrophils.

**FIG 7:**
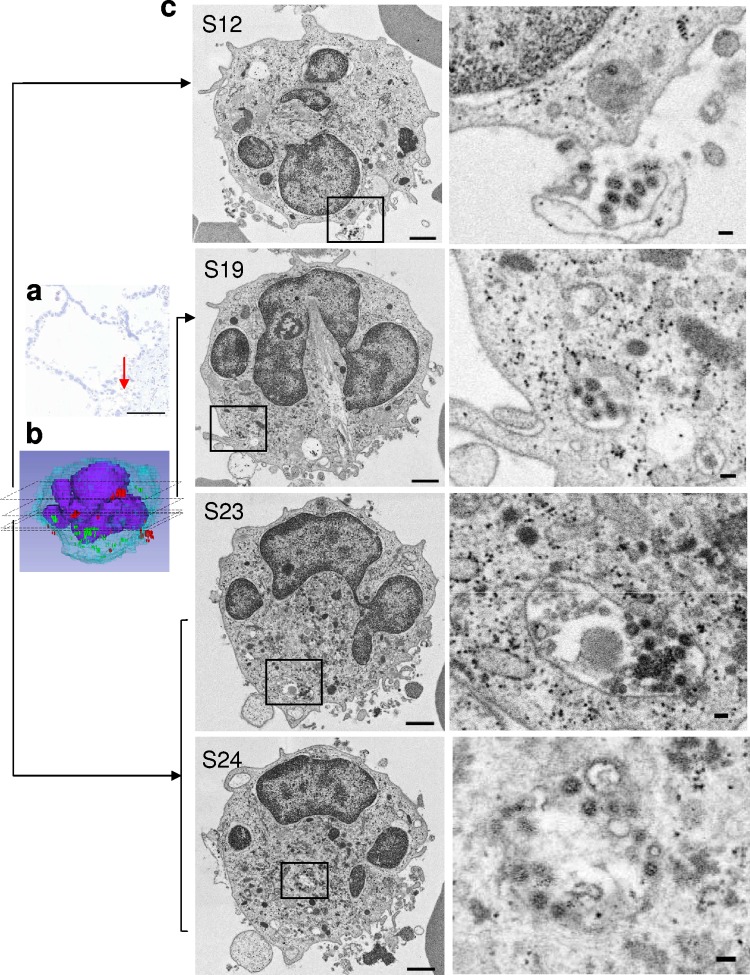
A single whole Neu in the alveolar space (red arrow) was analyzed by SSA-SEM. (a) Light microscopy of an alveolar section stained with toluidine blue showing the location of a Neu (red arrow). Scale bar = 100 μm. (b) Static 3D image of Neu-1. (c) Representative sections (S12, S19, S23, and S24) from 38 consecutive sections (each 200 nm thick) of Neu-1. The boxed areas (left) are shown at higher magnification on the right. Spherical virus particles are seen around the cell surface (S12). S19, S23, and S24 show a vesicle that contains several virus particles. (Left) Scale bars = 1 μm. (Right) scale bars = 100 nm.

Among the 100 serial sections used for SSA-SEM imaging, Neus were less common than Ms/Mϕs. Thus, three randomly selected Neus were analyzed by SSA-SEM (Tables 2 and 3). Virus particles were observed around the surfaces of Neu-1 and Neu-2, and intravesicular virus particles were found in Neu-1 (Tables 2 and 3). Neu-1 harbored several spherical virus particles within large vesicles (Fig. 7). No intranuclear dense tubules were seen in any of the 100 SSA-SEM images from the three whole cells examined (Table 2).

### 3D Visualization of SSA-SEM images.

**FIG 8:**
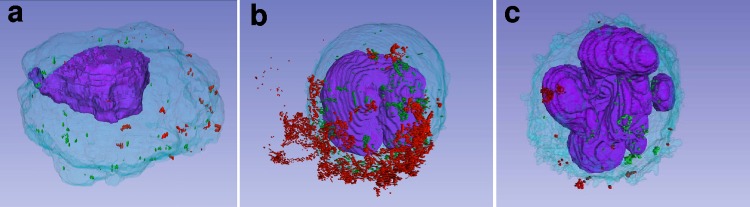
Representative static image of a 3D model of AEC-II-6 (a), M/Mϕ-2 (b), and Neu-1 (c) (Tables 2 and 3). Cytoplasm, light blue; nucleus, purple; virus particles within the cell, green; virus particles outside the cell membrane, red.

Specific structures, including cytoplasm (light blue), nucleus (purple), virus particles within the cell (green), and virus particles outside the cell (red), in each SSA-SEM image from serial sections were traced manually (Fig. 3). All SSA-SEM images were imported into 3D Slicer software for 3D visualization. The distribution of virus particles within desquamated AEC-II-6 (Fig. 5b and 8a; see Movie S1 in the supplemental material), M/Mϕ-2 (Fig. 6b, and 8b; see Movie S2 in the supplemental material), and Neu-1 (Fig. 7b and 8c; see Movie S3 in the supplemental material) was observed in the 3D images.

## DISCUSSION

EM enables visualization and identification of the morphology of virus particles, although it has a limited observable range and can provide only partial observation of large-scale structures. Most EM images of influenza virus particles have been captured using infected cultured cells (22–25). Several reports show TEM images of virus particles in autopsied human lung specimens, but the resolution of the images is limited and dependent on the condition of the specimens (18, 26–29). Here, we used TEM and SEM to observe virus particles and virus-related structures in autopsied lung specimens from a patient who had been infected with A/H1N1/pdm09 and subsequently died from viral pneumonia complicated by ARDS (8). A/H1N1/pdm09-D222G proliferated in the lung, and many viral antigens and much viral RNA were detected in AEC-IIs (Fig. 4f) (8, 10, 11). It should be noted that this study is based on limited analyses of a single autopsied human lung sample. Because sufficient virus particles remained in the sample, the novel SEM method was able to show the detailed distribution of virus particles and intranuclear dense tubules in AEC-IIs, Ms/Mϕs, and Neus.

Figure 3 shows how the same specimens were analyzed using TEM and the novel SEM method. The resolution of TEM images is higher than that of SEM images (Fig. 1 and 2), although the observation range is obstructed by the framework of the grid (Fig. 3). In contrast, specimens used for SEM are mounted on a glass slide, which enables stitching together of contiguous SEM images (Fig. 4a). By digitally stitching together SEM images, we observed an entire cross-section of a single alveolus and were able to identify virus particles and intranuclear dense tubules at high resolution. The stitched SEM image enables visualization from nanoscale to microscale within a single data set, making it a very useful tool for analyzing the distribution of virus particles and specific structures associated with viral infection.

In this study, we focused on AEC-IIs, Ms/Mϕs, and Neus and identified influenza virus particles and intranuclear dense tubules (M1-associated structures within the nucleus, which are a morphological characteristic of influenza virus-infected cells) in each cell type (16–20). The numbers of TEM and SSA-SEM images containing specific features and the numbers of virus particles and dense tubules in each cell are summarized in Tables 1 and 2 and in Table 3, respectively. It should be noted that all AEC-IIs in the tables were desquamated from the basement membrane due to the host immune response, suggesting arrest of viral replication. We think that this is why we saw no virus budding in any of the images (Table 1). Also, intravesicular virus particles were observed only in AEC-II-6 (Tables 2 and 3). This might be because influenza viruses fused rapidly in the cytoplasm after adsorption to the cells, making it difficult to capture images of intravesicular virus particles (30, 31). In contrast, a lot of dense tubules (M1 proteins) were observed in the nuclei of four of six AEC-IIs (Tables 2 and 3). This is consistent with a report that M1 proteins remain in the nucleus for a long time (30). Taken together, the results show that IAV infects AEC-IIs. In contrast, neither budding of IAV particles nor intranuclear dense tubules were observed in Ms/Mϕs and Neus, suggesting no IAV infection of these cells. However, four of six Ms/Mϕs and one of three Neus harbored many virus particles within vesicles (Tables 2 and 3).

We found no evidence that IAV replicated in Ms/Mϕs within the autopsied lung specimen. *In vitro* IAV infection of human Ms/Mϕs results in productive infection and release of infectious particles (32, 33) but can also result in abortive infection (34, 35). The results of infection may depend upon the particular subtype or strain of virus involved, as well as on the subset of Ms/Mϕs (36–38).

There are least two mechanisms by which IAVs can enter cells. A spherical IAV may enter a host cell through clathrin-dependent receptor-mediated endocytosis (39, 40). In addition, IAVs can induce macropinocytosis as an alternative entry pathway, independent of viral morphology (41, 42). Here, SSA-SEM revealed the presence of many virus particles in vesicles and around the surfaces of Ms/Mϕs. In the 20th SSA-SEM image of M/Mϕ-2, multiple virus particles, including filamentous viruses, were observed in the extracellular region in which the cell membrane entered the cytoplasm; this suggests induction of micropinocytosis (Fig. 6c). Furthermore, 3D reconstruction revealed that many virus particles were located on one side of the M/Mϕ-2 surface (Fig. 8b). Further investigation is required to determine whether this arrangement was coincidental or related to the functions of the M/Mϕ.

This study has several limitations. First the sample examined was obtained from a single human case. Second, virus particles and intranuclear dense tubules were identified morphologically but not confirmed by immunoelectron microscopy (immuno-EM). Unfortunately, we have no remaining samples available for immuno-EM studies. Since the specimens used for EM had been fixed with glutaraldehyde and osmium, their antigenicity has been destroyed. However, we point out that this is a common limitation of previous studies reporting EM images of influenza virus particles in human tissue (18, 26–29). There are no studies reporting immuno-EM of influenza virus in human lung tissue. Third, because autopsied specimens were obtained on day 7 post-disease onset, most of the AECs were desquamated from the basement membrane due to the host immune response. Therefore, evaluation of negative findings in AEC-IIs was difficult. Finally, we did not capture images of virus budding. Capturing images of virus budding is extremely difficult; indeed, no such images in human lung tissue have been reported to date. Acquisition of such images in postmortem lung tissue is dependent on the time after death and on the site of the sample used for EM analysis.

Despite being based on a single human case, these viruses were observed in postmortem influenza virus-infected human tissues. Conducting studies using postmortem biopsied lung tissue that is appropriate for image analysis of viral particles is extremely difficult. Thus, previous EM analyses of influenza viruses were conducted mainly in animal models or cultured cells. Despite the difficulties, we succeeded in obtaining clear images of the virus in human lung tissue.

Additionally, the study used novel experimental methods that are unprecedented in the field of influenza research. First, we successfully obtained EM images of an entire alveolus, which can be viewed at low and high magnification, by stitching together SEM images. This technique enabled visualization of both virus particles and intranuclear dense tubules (M1 proteins) in a single EM image. Second, 3D reconstruction of SSA-SEM images of an AEC-II, a M/Mϕ, and a Neu revealed virus distribution within a single whole cell. Thus, the analysis provides insight that could not be obtained by conventional 2D imaging.

This is the first report of the use of novel SEM methods to examine the distribution of IAV particles throughout a single whole cell in sections from an autopsy specimen. The differences between AEC-IIs, Ms/Mϕs, and Neus in terms of distribution of virus particles and virus-related structures suggest that each cell plays a different role in A/H1N1/pdm09 pneumonia.

## MATERIALS AND METHODS

### The patient.

The subject of the study was a 33-year-old man who died of respiratory failure resulting from A/H1N1/pdm09 pneumonia complicated by development of ARDS on the 7th day after onset of disease. An autopsy was carried out 3 h after death. The detailed pathological and molecular-biological findings have been reported (8–11). This study was approved by the institutional medical ethical committee of the National Institute of Infectious Diseases, Japan (approval no. 247).

### Fixation and tissue processing.

To prepare the specimens for EM, small parts of the lung tissue were excised and prefixed with 2% glutaraldehyde and 2.5% paraformaldehyde in 0.1 M phosphate buffer, pH 7.4, for 2 h at 4°C and then postfixed in 1% osmium tetroxide, dehydrated through a graded series of alcohols and propylene oxide, and embedded in epoxy resin. Other lung tissue specimens were routinely fixed in 10% buffered formalin and embedded in paraffin to prepare them for optical microscopy. Hematoxylin-eosin staining was performed for histological analysis, and immunohistochemistry was performed to evaluate the IAV nucleoprotein antigen, as previously described (8, 10, 11).

### Sectioning for TEM and SEM.

After trimming the epoxy resin-embedded tissue, ultrathin sections 70 nm thick were cut using an ultramicrotome (EM UC 6; Leica, Wetzlar, Germany), mounted on grids, and stained with 4% uranyl acetate and lead citrate for TEM using an H-7650 or an HT7700 (Hitachi, Tokyo, Japan). For SEM, ultrathin sections (200 nm) were cut using an ultramicrotome fitted with a histo-Jumbo diamond knife (DiAtome, Biel, Switzerland). Serial sections were placed on platinum-palladium-coated (MC1000; Hitachi, Tokyo, Japan) glass slides and allowed to adhere at 55°C for 30 min. The sections were stained with 1% toluidine blue for light microscopy and scanned with a NanoZoomer 2.0-RS digital slide scanner (Hamamatsu Photonics, Shizuoka, Japan). The observation regions were selected using NDP.view2 viewing software (Hamamatsu Photonics, Shizuoka, Japan). The sections were stained with 1% uranyl acetate and lead citrate and observed by ultra-high-resolution field emission SEM (SU8010; Hitachi, Tokyo, Japan). Regions of the ultrathin sections were imaged using backscattered electrons with an accelerating voltage of 1.5 kV. Images were taken at magnifications of ×7,000 to ×9,000, with a working distance of 3 mm. The workflows are shown in Fig. 3.

### Stitching together the SEM images.

To observe a whole cross-section of one alveolus, a 10-by-14 array of images was taken under the following conditions: accelerating voltage, 1.5 kV; working distance, 3 mm; magnification, ×5,000. After acquisition, the images were automatically aligned using the image stitching plugin in Fiji/Image J software (https://imagej.net/Image_Stitching) and viewed with the NDP.view2 viewing software. The workflows are shown in Fig. 3.

### 3D reconstruction of SSA-SEM images.

Serial section images were aligned automatically using the StackReg plugin in Fiji/Image J (https://imagej.net/StackReg). The aligned images were carefully checked visually and corrected manually if necessary. Subsequently, they were imported into Photoshop software (Photoshop CS5.1; Adobe Systems, San Jose, CA, USA), and the cytoplasm, nucleus, and viruses were manually selected by tracing their boundaries. After identification of the target structures in the serial images, 3D surface-rendering reconstruction was performed in 3D Slicer (https://www.slicer.org/).

## Supplementary Material

Supplemental file 1

Supplemental file 2

Supplemental file 3

Supplemental file 4

## References

[1] Noda T 2012 Native morphology of influenza virions. Front Microbiol 2:269. doi:10.3389/fmicb.2011.00269. 22291683 PMC3249889

[2] Nayak DP, Balogun RA, Yamada H, Zhou ZH, Barman S 2009 Influenza virus morphogenesis and budding. Virus Res 143:147–161. doi:10.1016/j.virusres.2009.05.010. 19481124 PMC2730999

[3] Shinya K, Ebina M, Yamada S, Ono M, Kasai N, Kawaoka Y 2006 Avian flu: influenza virus receptors in the human airway. Nature 440:435–436. doi:10.1038/440435a. 16554799

[4] Perez-Padilla R, de la Rosa-Zamboni D, Ponce de Leon S, Hernandez M, Quiñones-Falconi F, Bautista E, Ramirez-Venegas A, Rojas-Serrano J, Ormsby CE, Corrales A, Higuera A, Mondragon E, Cordova-Villalobos JA, INER Working Group on Influenza. 2009 Pneumonia and respiratory failure from swine-origin influenza A (H1N1) in Mexico. N Engl J Med 361:680–689. doi:10.1056/NEJMoa0904252. 19564631

[5] Mak GC, Au KW, Tai LS, Chuang KC, Cheng KC, Shiu TC, Lim W 2010 Association of D222G substitution in haemagglutinin of 2009 pandemic influenza A (H1N1) with severe disease. Euro Surveill 15:19534. 20394715

[6] Chan PK, Lee N, Joynt GM, Choi KW, Cheung JL, Yeung AC, Lam P, Wong R, Leung BW, So HY, Lam WY, Hui DC 2011 Clinical and virological course of infection with haemagglutinin D222G mutant strain of 2009 pandemic influenza A (H1N1) virus. J Clin Virol 50:320–324. doi:10.1016/j.jcv.2011.01.013. 21330192

[7] Ruggiero T, De Rosa F, Cerutti F, Pagani N, Allice T, Stella ML, Milia MG, Calcagno A, Burdino E, Gregori G, Urbino R, Di Perri G, Ranieri MV, Ghisetti V 2013 A/H1N1/pdm09 hemagglutinin D222G and D222N variants are frequently harbored by patients requiring extracorporeal membrane oxygenation and advanced respiratory assistance for severe A/H1N1/pdm09 infection. Influenza Other Respir Viruses 7 1416–1426. doi:10.1111/irv.12146. 23927713 PMC4634302

[8] Nakajima N, Hata S, Sato Y, Tobiume M, Katano H, Kaneko K, Nagata N, Kataoka M, Ainai A, Hasegawa H, Tashiro M, Kuroda M, Odai T, Urasawa N, Ogino T, Hanaoka H, Watanabe M, Sata T 2010 The first autopsy case of pandemic influenza (A/H1N1pdm) virus infection in Japan: detection of a high copy number of the virus in type II alveolar epithelial cells by pathological and virological examination. Jpn J Infect Dis 63:67–71. 20093768

[9] Kuroda M, Katano H, Nakajima N, Tobiume M, Ainai A, Sekizuka T, Hasegawa H, Tashiro M, Sasaki Y, Arakawa Y, Hata S, Watanabe M, Sata T 2010 Characterization of quasispecies of pandemic 2009 influenza A virus (A/H1N1/2009) by de novo sequencing using a next-generation DNA sequencer. PLoS One 5:e10256. doi:10.1371/journal.pone.0010256. 20428231 PMC2859049

[10] Nakajima N, Sato Y, Katano H, Hasegawa H, Kumasaka T, Hata S, Tanaka S, Amano T, Kasai T, Chong J-M, Iizuka T, Iiduka T, Nakazato I, Hino Y, Hamamatsu A, Horiguchi H, Tanaka T, Hasegawa A, Hasagawa A, Kanaya Y, Oku R, Oya T, Sata T 2012 Histopathological and immunohistochemical findings of 20 autopsy cases with 2009 H1N1 virus infection. Mod Pathol 25:1–13. doi:10.1038/modpathol.2011.125. 21874012

[11] Hayashi K, Yoshida H, Sato Y, Tobiume M, Suzuki Y, Ariyoshi K, Hasegawa H, Nakajima N 2017 Histopathological findings of lung with A/H1N1pdm09 infection-associated acute respiratory distress syndrome in the post-pandemic season. Jpn J Infect Dis 70:197–200. doi:10.7883/yoken.JJID.2016.120. 27357984

[12] Wacker I, Schroeder RR 2013 Array tomography. J Microsc 252:93–99. doi:10.1111/jmi.12087. 24111814

[13] Dittmayer C, Völcker E, Wacker I, Schröder RR, Bachmann S 2018 Modern field emission scanning electron microscopy provides new perspectives for imaging kidney ultrastructure. Kidney Int 94:625–631. doi:10.1016/j.kint.2018.05.017. 30143069

[14] Reichelt M, Joubert L, Perrino J, Koh AL, Phanwar I, Arvin AM 2012 3D reconstruction of VZV infected cell nuclei and PML nuclear cages by serial section array scanning electron microscopy and electron tomography. PLoS Pathog 8:e1002740. doi:10.1371/journal.ppat.1002740. 22685402 PMC3369938

[15] Koga D, Kusumi S, Ushiki T 2016 Three-dimensional shape of the Golgi apparatus in different cell types: serial section scanning electron microscopy of the osmium-impregnated Golgi apparatus. Microscopy 65:145–157. doi:10.1093/jmicro/dfv360. 26609075

[16] Gómez-Puertas P, Albo C, Pérez-Pastrana E, Vivo A, Portela A 2000 Influenza virus matrix protein is the major driving force in virus budding. J Virol 24:11538–11547. doi:10.1128/JVI.74.24.11538-11547.2000. PMC112434 11090151

[17] Josset L, Frobert E, Rosa-Calatrava M 2008 Influenza A replication and host nuclear compartments: many changes and many questions. J Clin Virol 43:381–390. doi:10.1016/j.jcv.2008.08.017. 18926763

[18] Goldsmith CS, Metcalfe MG, Rollin DC, Shieh WJ, Paddock CD, Xu X, Zaki SR 2011 Ultrastructural characterization of pandemic (H1N1) 2009 virus. Emerg Infect Dis 17:2056–2059. doi:10.3201/eid1711.110258. 22099097 PMC3310559

[19] Terrier O, Moules V, Carron C, Cartet G, Frobert E, Yver M, Traversier A, Wolff T, Riteau B, Naffakh N, Lina B, Diaz JJ, Rosa-Calatrava M 2012 The influenza fingerprints: NS1 and M1 proteins contribute to specific host cell ultrastructure signatures upon infection by different influenza A viruses. Virology 432:204–218. doi:10.1016/j.virol.2012.05.019. 22770924

[20] Terrier O, Carron C, Cartet G, Traversier A, Julien T, Valette M, Lina B, Moules V, Rosa-Calatrava M 2014 Ultrastructural fingerprints of avian influenza A (H7N9) virus in infected human lung cells. Virology 456-457:39–42. doi:10.1016/j.virol.2014.03.013. 24889223

[21] Dadonaite B, Vijayakrishnan S, Fodor E, Bhella D, Hutchinson EC 2016 Filamentous influenza viruses. J Gen Virol 97:1755–1764. doi:10.1099/jgv.0.000535. 27365089 PMC5935222

[22] Barman S, Adhikary L, Kawaoka Y, Nayak DP 2003 Influenza A virus hemagglutinin containing basolateral localization signal does not alter the apical budding of a recombinant influenza A virus in polarized MDCK cells. Virology 305:138–152. doi:10.1006/viro.2002.1731. 12504548

[23] Noda T, Sagara H, Yen A, Takada A, Kida H, Cheng RH, Kawaoka Y 2006 Architecture of ribonucleoprotein complexes in influenza A virus particles. Nature 439:490–492. doi:10.1038/nature04378. 16437116

[24] Iwatsuki-Horimoto K, Horimoto T, Noda T, Kiso M, Maeda J, Watanabe S, Muramoto Y, Fujii K, Kawaoka Y 2006 The cytoplasmic tail of the influenza A virus M2 protein plays a role in viral assembly. J Virol 11:5233–5240. doi:10.1128/JVI.00049-06. PMC1472145 16699003

[25] Noda T, Murakami S, Nakatsu S, Imai H, Muramoto Y, Shindo K, Sagara H, Kawaoka Y 2018 Importance of the 1 + 7 configuration of ribonucleoprotein complexes for influenza A virus genome packaging. Nat Commun 9:54. doi:10.1038/s41467-017-02517-w. 29302061 PMC5754346

[26] Mauad T, Hajjar LA, Callegari GD, da Silva LF, Schout D, Galas FR, Alves VA, Malheiros DM, Auler JO Jr, Ferreira AF, Borsato MR, Bezerra SM, Gutierrez PS, Caldini ET, Pasqualucci CA, Dolhnikoff M, Saldiva PH 2010 Lung pathology in fatal novel human influenza A (H1N1) infection. Am J Respir Crit Care Med 181:72–79. doi:10.1164/rccm.200909-1420OC. 19875682

[27] Ru YX, Li YC, Zhao Y, Zhao SX, Yang JP, Zhang HM, Pang TX 2011 Multiple organ invasion by viruses: pathological characteristics in three fatal cases of the 2009 pandemic influenza A/H1N1. Ultrastruct Pathol 35:155–161. doi:10.3109/01913123.2011.574249. 21657817

[28] Basu A, Shelke V, Chadha M, Kadam D, Sangle S, Gangodkar S, Mishra A 2011 Direct imaging of pH1N1 2009 influenza virus replication in alveolar pneumocytes in fatal cases by transmission electron microscopy. J Electron Microsc (Tokyo) 60:89–93. doi:10.1093/jmicro/dfq081. 21257735 PMC7543230

[29] Bal A, Suri V, Mishra B, Bhalla A, Agarwal R, Abrol A, Ratho RK, Joshi K 2012 Pathology and virology findings in cases of fatal influenza A H1N1 virus infection in 2009-2010. Histopathology 60:326–335. doi:10.1111/j.1365-2559.2011.04081.x. 22211291

[30] Martin K, Helenius A 1991 Transport of incoming influenza virus nucleocapsids into the nucleus. J Virol 65:232–244. 1985199 10.1128/jvi.65.1.232-244.1991 PMC240510

[31] Lakadamyali M, Rust MJ, Babcock HP, Zhuang X 2003 Visualizing infection of individual influenza viruses. Proc Natl Acad Sci U S A 100:9280–9285. doi:10.1073/pnas.0832269100. 12883000 PMC170909

[32] Yu WC, Chan RW, Wang J, Travanty EA, Nicholls JM, Peiris JS, Mason RJ, Chan MC 2011 Viral replication and innate host responses in primary human alveolar epithelial cells and alveolar macrophages infected with influenza H5N1 and H1N1 viruses. J Virol 85:6844–6855. doi:10.1128/JVI.02200-10. 21543489 PMC3126566

[33] Hoeve MA, Nash AA, Jackson D, Randall RE, Dransfield I 2012 Influenza virus A infection of human monocyte and macrophage subpopulations reveals increased susceptibility associated with cell differentiation. PLoS One 7:e29443. doi:10.1371/journal.pone.0029443. 22238612 PMC3251590

[34] Tate MD, Pickett DL, van Rooijen N, Brooks AG, Reading PC 2010 Critical role of airway macrophages in modulating disease severity during influenza virus infection of mice. J Virol 84:7569–7580. doi:10.1128/JVI.00291-10. 20504924 PMC2897615

[35] Friesenhagen J, Boergeling Y, Hrincius E, Ludwig S, Roth J, Viemann D 2012 Highly pathogenic avian influenza viruses inhibit effective immune responses of human blood-derived macrophages. J Leukoc Biol 92:11–20. doi:10.1189/jlb.0911479. 22442495 PMC3382316

[36] Marvin SA, Russier M, Huerta CT, Russell CJ, Schultz-Cherry S 2017 Influenza virus overcomes cellular blocks to productively replicate, impacting macrophage function. J Virol 91:e01417-16. doi:10.1128/JVI.01417-16. 27807237 PMC5215328

[37] Cline TD, Beck D, Bianchini E 2017 Influenza virus replication in macrophages: balancing protection and pathogenesis. J Gen Virol 98:2401–2412. doi:10.1099/jgv.0.000922. 28884667 PMC5725990

[38] Short KR, Brooks AG, Reading PC, Londrigan SL 2012 The fate of influenza A virus after infection of human macrophages and dendritic cells. J Gen Virol 93:2315–2325. doi:10.1099/vir.0.045021-0. 22894921

[39] Rust MJ, Lakadamyali M, Zhang F, Zhuang X 2004 Assembly of endocytic machinery around individual influenza viruses during viral entry. Nat Struct Mol Biol 11:567–573. doi:10.1038/nsmb769. 15122347 PMC2748740

[40] Fontana J, Steven AC 2015 Influenza virus-mediated membrane fusion: structural insights from electron microscopy. Arch Biochem Biophys 581:86–97. doi:10.1016/j.abb.2015.04.011. 25958107 PMC4543556

[41] de Vries E, Tscherne DM, Wienholts MJ, Cobos-Jiménez V, Scholte F, García-Sastre A, Rottier PJ, de Haan CA 2011 Dissection of the influenza A virus endocytic routes reveals macropinocytosis as an alternative entry pathway. PLoS Pathog 7:e1001329. doi:10.1371/journal.ppat.1001329. 21483486 PMC3068995

[42] Rossman JS, Leser GP, Lamb RA 2012 Filamentous influenza virus enters cells via macropinocytosis. J Virol 86:10950–10960. doi:10.1128/JVI.05992-11. 22875971 PMC3457176

