# Supplementary material for: Serial Section Array Scanning Electron Microscopy Analysis of Cells from Lung Autopsy Specimens following Fatal A/H1N1 2009 Pandemic Influenza Virus Infection
Source: J Virol. 2019 Sep 12;93(19):e00644-19. doi: 10.1128/JVI.00644-19 (PMC6744253; doi:10.1128/JVI.00644-19)
Supplement: Supplemental file 1 [file JVI.00644-19-s0004.pdf]

SUPPLEMENTARY MOVIE LEGENDS

Movie S1. 3D images of a type II alveolar epithelial cell (AEC-II) -6 in Table 2 and Table 3 showing the cytoplasm (light blue), nucleus (purple), virus particles in the cell (green), and virus particles outside the cell membrane (red). Virus particles were identified by the presence of spikes and capsids.

Movie S2. 3D images of a monocyte/macrophage (M/MΦ) -2 in Table 2 and Table 3 showing the cytoplasm (light blue), nucleus (purple), virus particles in the cell (green), and virus particles outside the cell membrane (red). Virus particles were identified by the presence of spikes and capsids.

Movie S3. 3D images of a neutrophil (Neu) -1 in Table 2 and Table 3 showing the cytoplasm (light blue), nucleus (purple), virus particles in the cell (green), and virus particles outside the cell membrane (red). Virus particles were identified by the presence of spikes and capsids.
